# Tobacco farming in rural Vietnam: questionable economic gain but evident health risks

**DOI:** 10.1186/1471-2458-9-24

**Published:** 2009-01-20

**Authors:** Hoang Van Minh, Kim Bao Giang, Nguyen Ngoc Bich, Nguyen Thanh Huong

**Affiliations:** 1 Faculty of Public Health, Hanoi Medical University Vietnam, Hanoi, Vietnam; 2 Department of Occupational Health, Hanoi School of Public Health, Hanoi, Vietnam; 3 Department of Health Education, Hanoi School of Public Health, Hanoi, Vietnam

## Abstract

**Background:**

In order to provide evidence on health impacts of the tobacco industry on cultivators in Vietnam, this study aims to provide comparison between tobacco cultivation related revenue and expenditure in selected areas in rural Vietnam and examine the relationship between tobacco cultivation and self-reported illness in the study population.

**Methods:**

Two tobacco farming communes and two non-tobacco farming communes were selected for this study. In each selected commune, 120 households were sampled using two-stage cluster sampling technique. Local health workers were recruited and trained to conduct household interviews using structured questionnaire.

**Results:**

Where the expenditure figures do not include personnel costs (as the farming work was almost always responsible by the family members themselves), it appeared that the average tobacco farmer did benefit financially from tobacco cultivation. However, if a personal opportunity cost was added to give a financial value to their labour, the profit from tobacco cultivation was seen to be minimal. The occurrences of 9 out of the 16 health problems were statistically significant higher among tobacco growing farmers compared to that among non-tobacco farmers. Tobacco farming was shown to be the second strong predictor of self-reported health problems among the farmer (after the effect of old age).

**Conclusion:**

The present study provides evidence that can be used to increase public awareness about the harmful effects of tobacco growing.

## Background

For years, in search of even more profits, the tobacco industry has encouraged countries and farmers to grow more tobacco. Tobacco companies have promoted tobacco growing as a panacea, claiming that it will bring unparalleled prosperity to farmers, their communities, and their countries [1].

Viet Nam is a prime target for the tobacco industry: a developing country with a tropical climate appropriate for tobacco cultivation, and hard-working laborers. The total area devoted to tobacco cultivation in Vietnam in 2002 was about 18,000 hectares (accounting for 0.28% of total agricultural land) which gave an output of about 27,400 tones of tobacco per year [2]. The number of full-time equivalent tobacco cultivators was about 136,000. The tobacco industry has established a plan to gradually increase domestic tobacco leaf production toward the year 2010 through increased production areas and improved yields [3].

While the cigarette industry argues that tobacco farming is a major contributor to the country's economy, the seriously damaging health and environmental impacts caused by tobacco farming have been well documented. From the moment the tobacco seed is planted to the time the tobacco plant is harvested and cured, the health of those who cultivate the crop is constantly at risk [1,2].

The hazards posed by tobacco cultivation place tobacco workers at increased risk of injury and illness. Children and adults (mainly women) working with tobacco frequently suffer from green tobacco sickness (GTS), which is caused by dermal absorption of nicotine from contact with wet tobacco leaves. GTS is characterized by symptoms that may include nausea, vomiting, weakness, headache, dizziness, abdominal cramps, and difficulty in breathing, as well as fluctuations in blood pressure and heart rate [4-6]. Large and frequent applications of pesticides to protect the plant from insects and diseases can cause poisoning, skin and eye irritation and other disorders of the nervous, respiratory systems, as well as kidney damage [7,8].

Tobacco growing also causes a lot of damage to the environment. In many developing countries wood is used as fuel to cure tobacco leaves and to construct curing barns. An internationally estimated 200 000 hectares of forests and woodlands are cut down each year because of tobacco farming [9]. Environmental degradation is also caused by the tobacco plant, which leaches nutrients from the soil, as well as pollution from pesticides and fertilizers applied to tobacco fields [10].

In Vietnam, tobacco control has recently received greater attention. The Vietnamese Government's readiness to curb the epidemic of tobacco related disease was reflected in the Prime Minister's Decision No 77/2002/QD-TTg on the Ratification of the Programme of Prevention and Control of Certain Non-communicable Diseases for the Period 2002–2010 [11] and the Government Resolution No 12/2000/NQ-CP on National Tobacco Control Policy 2000 – 2010 [12]. Vietnam signed the Framework Convention on Tobacco Control on August 8, 2003 and ratified it on 17 December 2004.

In order to enforce the policies on tobacco control in Vietnam, especially the enactment of the tobacco control law, reliable information on the economic and health effects of tobacco farming is urgently needed by health advocates, as well as for society in general. However, even though the amount of research on tobacco in Vietnam has recently increased rapidly, to the best of our knowledge, there remains no research on the health impact of the tobacco industry on cultivators. This study therefore aims to 1) provide a preliminary comparison between tobacco cultivation related revenue and expenditure in selected areas in rural Vietnam; and 2) examine the relationship between tobacco cultivation and self-reported illness in the study population. The findings of this study may be of use for evidence-based policy making against tobacco in Vietnam and elsewhere.

## Methods

### Study design and study site

This was a cross-sectional household survey. The study was undertaken in 2007 in 2 rural districts in Vietnam (Vo Nhai in the North and Cam My in the South). Vo Nhai district is located about 90 km north of Hanoi capital. The district has 14 communes and one town. It covers an area of about 85,000 hectares, mainly highland and mountainous areas. The total population of Vo Nhai in 2006 was about 63,000 people. Cam My district is located about 100 km south of Ho Chi Minh City. The district has 13 communes and 1 town, spread over 47,000 hectares. The total population of Cam My in 2006 was about 156,000. In both districts, tobacco cultivation has been clustered in several communes. The tobacco cultivation includes different types of work like land preparation, seeding/planting, taking care of the leaves, harvesting, curing/toasting, processing, storing, etc.

Two tobacco farming communes (one per study district) were selected for exposed subjects. We also chose two non-tobacco farming communes (one in each district and was similar to the exposed one in terms of geographical and demographic characteristics) for comparison. The non-tobacco farming communes were selected based on consultations with health bureau and health statistics office in the respective study district.

### Study sample and participants

**Figure 1:**
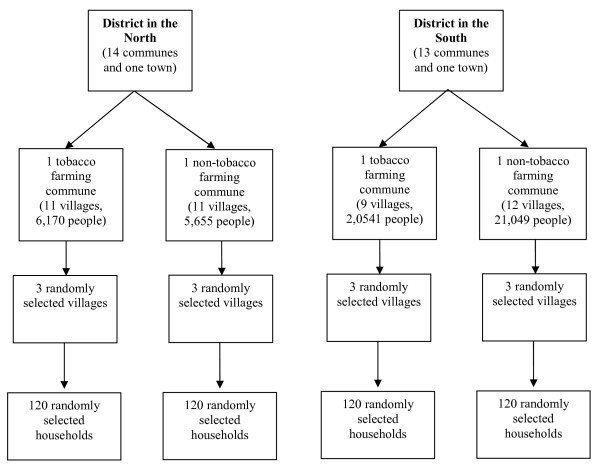
**Sampling procedure**.

In each selected commune, 120 households were sampled using two-stage cluster sampling technique. The sampling procedure is presented in Figure 1. The head of household was first interviewed about the family's livelihood (including information revenue and expenditure related to tobacco cultivation), then all other family members, aged 15–69 years old, were interviewed on the occurrence of illness during the last 6 months.

### Data collection

Local health workers were recruited and trained to conduct household interviews using structured questionnaire. The questionnaire was developed by research team with reference to the one used in the Vietnam Living Standard Survey 2002. It was pilot-tested in both the North and the South before official use. The field manual was also developed to ensure the standard of the data collection process. Spot-checks and re-checks of 10% sample data were conducted by the research team for quality control.

### Measurements

In this paper, tobacco cultivation-related revenue, expenditure and self-reported illness are the main outcome variables. Information on tobacco cultivation-related revenue and expenditure was obtained from detailed interviews with the heads of household. The annual revenue from tobacco cultivation is the total amount of money the family gets from the sales of all tobacco products (fresh, cured tobacco leaves, hand rolled cigarettes, etc.) produced in a year. The annual expenditure on tobacco production is the sum of different items needed for the whole process (land preparation, seeding/planting, taking care of the leaves, harvesting, curing/toasting, processing, and storing, etc.). There were 9 cases where the respondents did not remember an input quantity and/or price, estimates based on corresponding figures provided by neighbors were used to calculate the expenditure.

Information on self-reported illness during the last six months among the study populations was collected using questions about the occurrence of 16 health problems (Table 1). The inclusion of these 16 health problems was based on the advice from experts and results of the pilot study. The response set was a five-point scale where 1 = never, 2 = rarely, 3 = sometimes, 4 = often, 5 = always. The reliability in terms of internal consistency among the 16 illnesses/symptoms items, as measured by Cronbach's Alpha coefficient, was good (α = .83) [13]. Two composite indices were constructed from the 16 questions. The first one, called "illness presence", is a dichotomous variable in which "yes" denotes the occurrence any of 16 selected health problems. The second one, called "total illness score", is a continuous variable, which was calculated by the summation of the points of all the 16 scales.

**Table 1:** Self-reported illness among study populations during the last 6 months

**No**	**Symptoms**	**Tobacco farmers** **n(%)**	**Non-tobacco farmers** **n(%)**
**1**.	**Tiredness/weakness**	434 (90.0)***	**372 (76.5)**

**2**.	**Nausea**	139 (28.8)***	**92 (18.9)**

3.	Vomiting	52 (10.8)	62 (12.8)

4.	Dizziness	283 (58.7)	307 (63.2)

5.	Headache	374 (77.6)	352 (72.4)

6.	Abdominal pain	135 (28.0)	166 (34.2)

7.	Insomnia	271 (56.2)	245 (50.4)

8.	Difficult breathing/shortness of breath	117 (24.3)	102 (21.0)

**9**.	**Increased perspiration/sweating**	321 (66.6)***	**134 (27.6)**

**10**.	**Chill**	99 (20.5)***	**56 (11.5)**

**11**.	**High heart rate**	129 (26.8)*	**98 (20.2)**

12.	Pallor	84 (17.4)	65 (13.4)

**13**.	**Increased salivation**	59 (12.2)*	**38 (7.8)**

**14**.	**Whole body dull pain**	414 (85.9)*	**388 (79.8)**

**15**.	**Poor appetite**	232 (48.1)***	**158 (32.5)**

**16**.	**Itchy, rushing**	113 (23.4)***	**68 (14.0)**

	**Total**	**482 (100)**	**486(100)**

*p < 0.05; *** p < 0.001

Tobacco cultivation status (yes/no) and socio-demographic conditions of the study participants were included as independent variables. The socio-demographic conditions of the study subjects were assessed by educational level, occupational status and per capita income per month. Information on education and occupation was obtained through the direct interviews with the study subject. Educational level was classified into five groups: (I) no education; (II) not yet complete primary education; (III) complete primary education (completion of grade 6); (IV) complete primary education (completion of grade 9); (V) tertiary education (completion of grade 12) and higher. Occupational status (main occupation of the study subjects) was grouped as: (I) farmer; (II) government staff; (III) pupil/student and (IV) other jobs (small traders, construction workers, handicraft makers, etc.). Economic status of the respondent's household was measured by income quintiles. Information on income was collected through detailed interviews with the head of household. Average per capita income per month was the total income of the household divided the number of household members.

### Data management and analysis

Data were processed using Epi-Data by experienced research assistants. Double entry was applied with 10% filled questionnaires. Both descriptive and analytical statistics were carried out using Stata 9 software (Stata Corporation). The Chi squared test was used to examine the differences in the occurrence of 16 illnesses/symptoms among the tobacco growers compared to that among the non-tobacco farmers. Multivariate logistic regression and linear regression modeling were performed to establish the relationships of "illness presence" and "total illness score" with tobacco cultivation status as well as the socio-demographic variables. Both logistic and linear regression models were constructed using fixed variable method (i.e. based on our hypothesis on the relationships between outcome variables and independent factors). A cluster option was introduced in the analyses to reflect the nature of the sampling technique. A significance level of p < 0.05 was used. In calculating expenditure and revenue, local currency values were converted into US dollars using the 2007 exchange rate of US$ 1 = VND 16,000.

### Ethical clearance

Ethical clearance for conducting this research was given by the Institutional Review Board of Hanoi School of Public Health. The study also got the approval from People's Commune Committees in each study commune. Before participating into this study, all invited respondents were provided with clear information regarding this research. They were informed that participation would be voluntary following informed consent. Their responses would be confidential, there would be no right or wrong answers, and they could stop or withdraw from participation at any time. The refusal or withdrawal would not have any effect on them.

## Results

### General description of the study populations

A total of 480 households from the four selected communes were surveyed. All the study communes had nearly the same percentage of men and women. A large proportion of population in the study communes aged below 44 years old and a small proportion of people were elderly (i.e. aged 65 year old and over). The educational level of the study populations was quite limited. The main occupation of the populations in the studied areas was recorded as 'farmer'. There was no significant difference in demographic characteristics between the tobacco farmers and the non tobacco-farming ones (Table 2).

**Table 2:** General socio-demographic characteristics of the study populations

**Characteristics**	**South**			**North**		
	
	Tobacco farming commune	Non-tobacco farming commune	p value	Tobacco farming commune	Non-tobacco farming commune	p value
**Sex: n (%)**						

▪ Men	286 (50.8)	273 (48.1)	0.36*	243 (48.4)	237 (48.7)	0.96

▪ Women	277 (49.2)	295 (51.9)		259 (51.6)	250 (51.3)	

						

**Age: n (%)**						

▪ <15	164 (29.1)	178 (31.3)	0.15*	115 (22.9)	113 (23.2)	0.84*

▪ 15–24	146 (25.9)	137 (24.1)		100 (19.9)	88 (18.1)	

▪ 25–44	136 (24.2)	127 (22.4)		173 (34.5)	181 (37.2)	

▪ 45–64	108 (19.2)	104 (18.3)		83 (16.5)	80 (16.4)	

▪ 64+	9 (1.6)	22 (3.9)		31 (6.2)	25 (5.1)	

						

**Education: n (%)**						

▪ No education	29 (5.2)	31 (5.5)	0.69*	10 (2.1)	17 (3.5)	0.06*

▪ Not yet complete primary level	112 (19.9)	111 (19.5)		63 (12.6)	94 (19.3)	

▪ Complete primary level	200 (35.5)	190 (33.5)		105 (20.9)	77 (15.8)	

▪ Complete secondary school	155 (27.5)	152 (26.8)		212 (42.2)	178 (36.6)	

▪ Tertiary education and higher	67 (11.9)	84 (14.8)		101 (20.1)	121 (24.9)	

						

**Occupation: n (%)**						

▪ Farmer	280 (49.7)	280 (49.3)	0.33*	330 (65.7)	279 (57.3)	0.01*

▪ Government staff	4 (0.7)	4 (0.7)		6 (1.2)	24 (4.9)	

▪ Pupil/student	211 (37.5)	195 (34.3)		122 (24.3)	132 (27.1)	

▪ Other	68 (12.1)	89 (15.7)		44 (8.8)	52 (10.7)	

						

**Per capita income: mean(sd) US$**	28.5 (24.0)	20.4 (15.6)	0.00**	19.1 (9.3)	21.8 (14.7)	0.00**

						

**Total**	**563 (100)**	**568 (100)**		**502 (100)**	**487 (100)**	

* p value for chi squared test

** p value for median test

However, there was variation in economic conditions across the four communes. The per capita income per month was highest in the tobacco-farming commune in the South (US$ 28.5) and lowest in the tobacco-farming commune in the North (US$ 19.1) (Table 2).

### Tobacco cultivation related expenditure and revenue

The figures on the amount of money each household spent a year on tobacco cultivation and the revenue the family got from the corresponding harvest are presented in Table 3. Where the expenditure figures do not include personnel costs (as the farming work was almost always responsible by the family members themselves), it appeared that the average tobacco farmer did benefit financially from tobacco cultivation (expenditure of US$ 238.8 vs. revenue of US$ 513.0).

**Table 3:** Tobacco cultivation related expenditure and revenue (in US$)

**Expenditure and Revenue**	**South**					**North**				
	
	mean	sd	median	min	max	mean	sd	median	min	max
Annual expenditure (personnel cost not included)	201.2	156.2	187.7	124.3	612.5	279.3	137.0	275.0	135.3	618.8

Annual expenditure (personnel cost included)	376.0	273.9	374.3	213.4	726.1	609.9	240.0	621.9	187.0	955.0

Annual revenue	553.4	434.5	500.0	323.2	850.0	467.6	290.3	437.5	233.8	997.0

However, if a personal opportunity cost was added to give a financial value to their labour (using a rate of US$2 per day as the accepted rate for manual labour), it seemed that tobacco farmers in the South got some profit from tobacco cultivation. However, the profit was seen to be minimal (expenditure of US$ 481.4 vs. revenue of US$ 513.0). In the tobacco farming commune in the North, including opportunity costs, the expenditure on tobacco cultivation was higher than the corresponding revenue (expenditure of US$ 609.9 vs. revenue of US$ 467.6).

### The association between tobacco cultivation and self-reported illness

In this study, a total of 968 farmers aged from 15 to 69 years old from the four selected communes (480 households) were interviewed about the occurrence of the 16 selected health problems. Table 1 presents the proportions of respondents who reported to have the problems during the last 6 months. The occurrences of 9 out of the 16 health problems were statistically significant higher among tobacco growing farmers compared to that among non-tobacco farmers.

The multivariate logistic regression analyses of the effects of tobacco cultivation as well as socio-demographic factors on "illness presence" are presented in Table 4. People who cultivated tobacco were 3.5 times more likely to have a health problem than those who did not (OR = 3.5; 95%CI = 1.5–8.0). The occurrence of a health problem significantly increased among people in the lower income quintiles.

**Table 4:** Multivariate logistic regression analyses of the effects of tobacco cultivation as well as socio-demographic factors on "illness presence".

**Illness presence (Yes/No)**	**Odds ratios (95%CI)**
**Tobacco cultivation**	

Yes	3.5 (1.5; 8.0)*

No	1

**Gender**	

Men	1

Women	1.5 (0.7; 3.1)

**Age**	

16–24	1

25–44	2.5 (0.4; 10.3)

44–69	2.9 (0.9; 11.0)

**Education**	

Less than tertiary education	1

Tertiary education and higher	1.1 (0.5; 2.3)

**Occupation**	

Farmer	1

Government staffs	0.5 (0.1; 2.0)

Other jobs	1.8 (0.3; 9.2)

**Income quintile**	

1st quintile	5.9 (1.6; 21.3)*

2nd quintile	5.0 (1.6; 15.7)*

3rd quintile	4.1 (1.3; 12.9)*

4th quintile	3.5 (1.2; 10.2)*

5th quintile	1

R-squared = 0.11*

* Denotes significant result

The effects of tobacco cultivation and socio-demographic variables on "total illness score" were examined by multivariate linear regression and shown in Table 5. The regression model shows that people who grew tobacco, older people, the women, and the individuals with lower economic status were more likely to have increased frequencies of the identified health problems. The difference in "total illness score" by economic status was statistically significant for those the first quintile and the second income quintile compared to those in the highest quintile. Table 5 also shows the standardized regression coefficients. Tobacco farming was shown to be the second strong predictor within the model (after effect of old age).

**Table 5:** Multivariate linear regression analyses of the effects of tobacco cultivation and socio-demographic status on "total illness score"

**Total illness score**	**Coefficients**	**SE**	**Standardized partial regression coefficient**
**Intercept**	23.57*	1.46	-

**Tobacco cultivation**	3.59 *	0.48	0.23

**Men**	2.60*	0.48	0.17

**Higher age**	0.03*	0.01	0.10

**Higher education**	-0.16	0.48	-0.01

**Occupation**	0.32	0.50	0.02

**Higher income quintile**	-0.78*	0.17	-0.14

R-squared = 0.11*

* Denotes significant result (p < 0.05)

## Discussions

While the economic and health problems associated with both active and passive tobacco smoking have been well documented in literature worldwide, little is known about the effects of tobacco cultivation, especially in developing countries [14]. The present study, which is among the first of this kind conducted in Vietnam, provides valuable evidence surrounding the socio-economic and health effects of tobacco growing in the Vietnamese context.

The demographic characteristics of the study populations are typical for rural communities in Vietnam. The education level is low, and farming is the predominant occupation. The distributions of age and sex in the population correspond well to the usual pattern of population pyramid in Vietnam, which has a small proportion of elderly people.

The figures of monthly income indicate that tobacco cultivators are not wealthier than other farmers (Table 2). This is contrary to the tobacco companies' claim that "tobacco brings prosperity to its planters" [15] and "tobacco is an important solution for hunger elimination and poverty reduction" [16]. A study from China also showed that tobacco cultivation brought lower returns than vegetable oil, beans, or fruit [17]. Similarly, the fact that tobacco farming had lower revenue-to-cost ratio than other crops has also been reported in studies from Kenya [18] and India [19]. A recent report by WHO also confirmed that tobacco growing entails a number of irreversible costs to farmers, including damage their living standards and erode their long-term prospects [20]. The finding of the relationship between tobacco farming related expenditure and revenue also confirms the fact that tobacco cultivation does not bring tangible economic gain to the tobacco planters. Higher benefit would be received if farmers had invested their time and resources in something else, or had been hired by others for manual labour, rather than investing in tobacco cultivation. The finding suggests that creating more jobs for local people, even manual labour, is financially competitive with growing tobacco, with its attendant health risks, discussed below.

Our data clearly show that tobacco cultivation was strongly associated with the occurrence of a range of health problems. The finding is similar to those reported by previous studies, conducted in other countries [14,21,22]. The health problems are known to be induced by direct contact with tobacco plants (nicotine poisoning), high levels of exposure to toxic pesticides and the physical consequences of hard labour [4-8,23]. The most controversial and serious environmental health issue in tobacco agriculture is pesticide use. Breathing high doses of pesticide can produce respiratory irritation, nausea, headache, and fatigue. It is estimated that 25 million pesticide poisonings occur every year in developing countries [24]. A study from Malaysia in 1995 already proved that tobacco are ate high risk of pesticide poisoning [25]. A study conducted by the Kenya Medical Research Institute reported 1,000 deaths and 35,000 cases of occupational poisoning on all farms in 1997[26]. In Brazil, 300,000 tobacco growers are poisoned from pesticide use annually [26]. In the United States, the National Institute for Occupational Safety and Health (NIOSH) estimates there are 10,000 physician-diagnosed pesticide poisonings annually [24].

The findings of the present study indicated that increasing age was associated with higher occurrence of tobacco farming related health problems (Table 5). This is different from the findings of previous international studies which reported that younger workers are more likely than older ones to develop GTS [4,23].

We found that the health problems were more commonly reported by the women than men (Table 5). This is also different from the pattern found in other international investigations which showed that nearly all of those affected by GTS are male [4,23]. One common element of the explanation for women's higher rates of morbidity is that there are gender differences in the way that symptoms are perceived, evaluated and acted upon. However, a study in rural Vietnam has shown no gender differences in the reporting of health problems [27]. This suggests that there may be gender inequality in the health effects of tobacco growing in Vietnam. In fact, it is important to note that the roles women are vital at almost all stages of tobacco farming in the study settings. Women not only share with men the role of economic producers though their labor, but do so under the added weight of their roles as biological producers of children and social reproducers through child-rearing and household management. Given the findings, actions toward women's livelihoods and health in the study settings are urgently needed.

The present study also revealed clear economic disparities in health effects of tobacco cultivation (Table 4, 5). The poor are proven to be more vulnerable to the harmful effects of tobacco growing. The poorer are known to be almost always more susceptible to illness[28,29], so they need to be better protected and supported by both social and health policies. In the context of this study, providing local people with more alternative earning opportunities would reduce the health inequality issue.

The study uses a retrospective approach to collect information on income, expenditure, and self-reported illness. This may be open to recall bias, especially information on annual income and expenditures on and details of pesticides, fertilizers, etc.

The validity of self reported information also depends on characteristics of both interviewers and respondents. Probing skills of interviewers are very important. In this study, village health workers were selected as interviewers because they already had some experiences in doing household interviews. However, this was the first time they did interviews using a long questionnaire with quite many difficult questions such as estimation of expenditure, revenue, name of fertilizer, pesticide, etc. Even though the trainings were conducted carefully, the interviewers still made a number of mistakes. As a result, about 10% of interviews were redone by researchers of this study.

Characteristics of respondents such as their educational level, their ability to recall it and their willingness to report it, might also have influenced the validity of the study findings. In this study, we had difficulties when asking the respondents, who were normally with low education, to recall the name of pesticides, fertilizer they used and make some calculations and estimations on quantity of pesticide, fertilizer used per unit of land, etc. As a result, the information collected might not be totally correct.

## Conclusion

Vietnam is still in the early stages of the battle against tobacco. The findings from the present study provide valuable and timely evidence that can be used to increase public awareness as well as develop and implement appropriate responses to the harmful effects of tobacco growing.

## Competing interests

The authors declare that they have no competing interests.

## Authors' contributions

Hoang Van Minh, Kim Bao Giang, Nguyen Ngoc Bich and Nguyen Thanh Huong made substantial contributions to conception and design, or acquisition of data, or analysis and interpretation of data. All three have been involved in drafting the manuscript or revising it critically for important intellectual content.

## Pre-publication history

The pre-publication history for this paper can be accessed here:



## References

[B1] Campaign for Tobacco Free Kids (2001). Golden leaf barren harvest, the costs of tobacco farming.

[B2] Mackay J, Eriksen M (2005). The Tobacco Atlas.

[B3] Kinh HV, Bales S (2002). Tobacco in Viet Nam:the industry, demand, control policies and employment.

[B4] Ballard T (1995). Green tobacco sickness: occupational nicotine poisoning in tobacco workers. Archives of Environmental Health.

[B5] Southeast Center Studies Ways To Prevent Green Tobacco Sickness (1996). NIOSH Agricultural Health & Safety Center News.

[B6] Arcury TA (2003). High levels of transdermal nicotine exposure produce green tobacco sickness in Latino farm workers. Nicotine & Tobacco Research.

[B7] Cox C (1992). 1,3 – Dichloropropene. Journal of Pesticide Reform.

[B8] Cox C (1995). Chlorpyrifos Factsheet, Part 2. Journal of Pesticide Reform.

[B9] Geist HJ (1999). Global assessment of deforestation related to tobacco farming. Tobacco Control.

[B10] Geist HJ, Lohnert B, Geist H (1999). Soil Mining and Societal Responses. Coping with Changing Environments.

[B11] Viet Nam Prime Minister's Office (2002). Decision 77/2002/QD-TTg: Ratification of Programme of Prevention and Control of Certain Noncommunicable Diseases for the Period 2002–2010.

[B12] Viet Nam Prime Minister's Office (2000). Government Resolution No.12/2000/NQ-CP on National Tobacco Control Policy 2000 – 2010.

[B13] Pallant J (2004). SPSS survival manual: a step by step guide to data analysis using SPSS.

[B14] McBride JS, Altman DG, Klein M, White W (1998). Green tobacco sickness. Tobacco Control.

[B15] Thang HD (2003). Investment in planting tobacco in Vietnam.

[B16] Ministry of Planning and Investemnt (2000). Situation of cigarrete trading in Vietnam 1999–2000.

[B17] Hu TW, Mao Z, Ong M, Tong E, Tao M, Jiang H, Hammond K, Smith KR, de Beyer J, Yurekli A (2006). China at the crossroads: the economics of tobacco and health. Tob Control.

[B18] Kweyuh PHM, Abedian I, van der Merwe R, Wilkins N, Jha P (1998). Does tobacco growing pay? The case of Kenya. The economics of tobacco control: towards an optimal policy mix.

[B19] Chari MS, Kameswara, Rao BV, Gupta PC, Hammer JE, Murti PR (1992). Role of tobacco in the national economy: past and present. Control of tobacco-related cancers and other diseases: international symposium 1990.

[B20] World Health Organization (2008). WHO Framework Convention on Tobacco Control. Conference of the Parties to the WHO Framework Convention on Tobacco Control Durban, South Africa.

[B21] Parikh JR, Gokani VN, Kulkarni PK, Shah AR, Saiyed HN (2005). Acute and Chronic Health Effects Due to Green Tobacco Exposure in Agricultural Workers. American Journal of Industrial Medicine.

[B22] Arcury TA, Quandt SA, JS P (2001). Predictors of incidence and prevalence of green tobacco sickness among Latino farmworkers in North Carolina, USA. Journal of Epidemiology Community Health.

[B23] McBride JS, Altman DG, Klein M, White W (1998). Green tobacco sickness.

[B24] Brown VJ (2003). Tobacco's profit, workers' loss?. Environ Health Perspect.

[B25] Cornwall JE, Ford ML, Liyanage TS, Daw DWK (1995). Risk assessment and health effects of pesticides used in tobacco farming in Malaysia.

[B26] Campaign for Tobacco-Free Kids (2001). Golden Leaf, Barren Harvest.

[B27] Giang KB, Allebeck P (2003). Self-reported illness and use of health services in a rural district of Vietnam: findings from an epidemiological field laboratory. Scand J Public Health Suppl.

[B28] Mackenbach JP (2002). Income inequality and population health.

[B29] Yiengprugsawan V, Lim L, Carmichael G, Sidorenko A, Sleigh A (2007). Measuring and decomposing inequity in self-reported morbidity and self-assessed health in Thailand.

